# Effect of amblyopia treatment on choroidal thickness in hypermetropic anisometropic amblyopia using swept-source optical coherence tomography

**DOI:** 10.1186/s12886-018-0894-z

**Published:** 2018-08-31

**Authors:** Syunsuke Araki, Atsushi Miki, Katsutoshi Goto, Tsutomu Yamashita, Go Takizawa, Kazuko Haruishi, Tsuyoshi Yoneda, Yoshiaki Ieki, Junichi Kiryu, Goro Maehara, Kiyoshi Yaoeda

**Affiliations:** 1 Department of Ophthalmology, Kawasaki Medical School, 577 Matsushima, Kurashiki, Okayama, 701-0192 Japan; 2 Department of Sensory Science, Faculty of Health Science and Technology, Kawasaki University of Medical Welfare, 288 Matsushima, Kurashiki, Okayama, 701-0193 Japan; 3 Department of Human Sciences, Kanagawa University, 3-27-1 Rokkakubashi, Yokohama, Kanagawa 221-8686 Japan; 4 Yaoeda Eye Clinic, 2-1649-1 Naga-Chou, Nagaoka, Niigata, 940-0053 Japan

**Keywords:** Amblyopia, Choroid, Treatment, Optical coherence tomography

## Abstract

**Background:**

Recent studies using optical coherence tomography (OCT) have indicated that choroidal thickness (CT) in the anisometropic amblyopic eye is thicker than that of the fellow and normal control eyes. However, it has not yet been established as to how amblyopia affects the choroid thickening. The purpose of the present study was to investigate the effect of amblyopia treatment on macular CT in eyes with anisometropic amblyopia using swept-source OCT.

**Methods:**

Thirteen patients (mean age: 6.2 ± 2.4 years) with hypermetropic anisometropic amblyopia were included in this study. Visual acuity (VA), axial length (AL), and CT were measured at the enrollment visit and at the final visit, after at least 6 months of treatment. CT measurements were corrected for magnification error and were automatically analyzed using built-in software and divided into three macular regions (subfoveal choroidal thickness (SFCT), center 1 mm, and center 6 mm). A one-way analysis of covariance using AL as a covariate was performed to determine whether CT in amblyopic eyes changed after amblyopia treatment.

**Results:**

The average observation period was 22.2 ± 11.0 months. After treatment, VA (logMAR) improvement in the amblyopic eyes was 0.41 ± 0.19 (*p* < 0.001). SFCT, center 1 mm CT, and center 6 mm CT were significantly thicker in the amblyopic eyes compared with the fellow eyes both before and after treatment (*p* < 0.05 for all comparisons). There were no significant changes in SFCT, center 1 mm CT, or center 6 mm CT before and after treatment in the amblyopic (*p* = 0.25, 0.21, and 0.84, respectively) and fellow (*p* = 0.75, 0.84, and 0.91, respectively) eyes. The correlation between changes in logMAR versus changes in CT after treatment was not significant.

**Conclusions:**

Although VA in amblyopic eyes was significantly improved after treatment, the choroid thickening of anisometropic amblyopic eyes persisted, and there was no significant change found in the CT after the treatment. Our findings suggest that thickening of the CT in amblyopia is not directly related to visual dysfunction.

## Background

Amblyopia is defined as a disorder in which there is dysfunction in processing visual information such as reduced recognition visual acuity (VA) [1]. The pathogenesis of amblyopia has been thought to be based on morphological and functional abnormalities in the visual cortex and lateral geniculate nucleus [2–5]. In contrast, it is unclear whether or not dysfunction or structural abnormality of the retina is present in amblyopia [6].

Recent studies using optical coherence tomography (OCT) have indicated that retinal or choroidal thickness (CT) in amblyopic eyes is thicker than in fellow and normal control eyes [7, 8]. However, since amblyopic eyes are often smaller than fellow eyes [9], it is almost impossible to find control subjects, as these small eyes are almost always hyperopic and normally amblyopic. Therefore, a consensus as to whether the retina or choroid thickening in amblyopic eyes is affected by the “pathologic condition of amblyopia” or a “difference in the ocular size” has yet to be established. In our previous study [10], we found there was no significant difference in the macular inner retinal thickness in unilateral amblyopia patients. In addition, although we found significant differences in CT in patients with hypermetropic anisometropic amblyopia, there was no significant difference found for strabismic amblyopia. The results of our previous study do not support the hypothesis that the choroid thickening is simply due to differences in ocular size, as we analyzed the choroidal thickness after adjusting for the axial length (AL). There must be another contributing factor, but it remains unclear as to how amblyopia is able to affect the choroid thickening.

A few recent studies researched the effect of amblyopia treatment on CT in order to investigate the relationship between amblyopia and choroid [11–14]. However, to the best of our knowledge, there have been no studies in which a swept-source OCT (SS-OCT) has been used to compare the CT before and after treatment. Furthermore, as SS-OCT uses a long-wavelength light source of 1 μm, utilizing this technique to examine the choroid can provide superior imaging as compared to spectral-domain OCT (SD-OCT). Thus, the purpose of the present study was to investigate the effect of amblyopia treatment on the macular CT in anisometropic amblyopia eyes using the SS-OCT technique.

## Methods

All of the investigative procedures used respect the Declaration of Helsinki and approval from the Institutional Review Board Committee of Kawasaki Medical School (registration number: 2458–1) was obtained. This study was designed as an observational case series and conducted from November 2013 to June 2017 in the Department of Ophthalmology at Kawasaki Medical School Hospital. Informed consent for the examinations was obtained from one of the parents of each patient.

### Subjects

This study enrolled 16 patients aged 4 to 12 years. All patients were diagnosed with hypermetropic anisometropic amblyopia, and underwent ophthalmologic examinations at the first visit including best-corrected VA (BCVA), cycloplegic refraction using an autorefractor (RKT-7700, NIDEK Co., Ltd., Gamagori, Japan), intraocular pressure, AL (IOL Master®, Carl Zeiss Meditec AG, Jena, Germany), cover test, extraocular movements, slit-lamp, funduscopy, and SS-OCT (DRI OCT-1 Atlantis®, Topcon Corporation, Tokyo, Japan).

The exclusion criteria were as follows: presence of BCVA better than 0.0 logMAR within two months after full refractive correction, history of amblyopia treatment, ocular diseases, history of intraocular surgery, presence of systemic diseases that may have had an influence on the eye, and an SS-OCT image in which the auto-segmentation was difficult to obtain due to signal attenuation.

Anisometropia was defined as an interocular difference in refraction (spherical equivalent) of more than 2.0 diopters (D). Hypermetropic anisometropic amblyopia was defined as the presence of a BCVA worse than 0.1 logMAR in the eye which had more diopters after full refractive correction.

Initially all patients received the full refractive correction. BCVA was measured at least every 1 to 2 months. After no further VA improvement, patients underwent additional patching treatment (2 to 6 h/day) [15]. Patching treatment was reduced or stopped once the patient achieved the maximum VA. BCVA, AL, and CT were measured at the enrollment visit and at the final visit after treatments of at least 6 months.

### CT measurements

CT measurements were performed using SS-OCT. SS-OCT parameters included a 1050 nm wavelength light source, depth resolution of 8.0 μm, and a scan speed of 100,000 A-scans/second. Data analysis was performed using the DRI OCT-1 Atlantis® software program version 9.30.

**Fig. 1:**
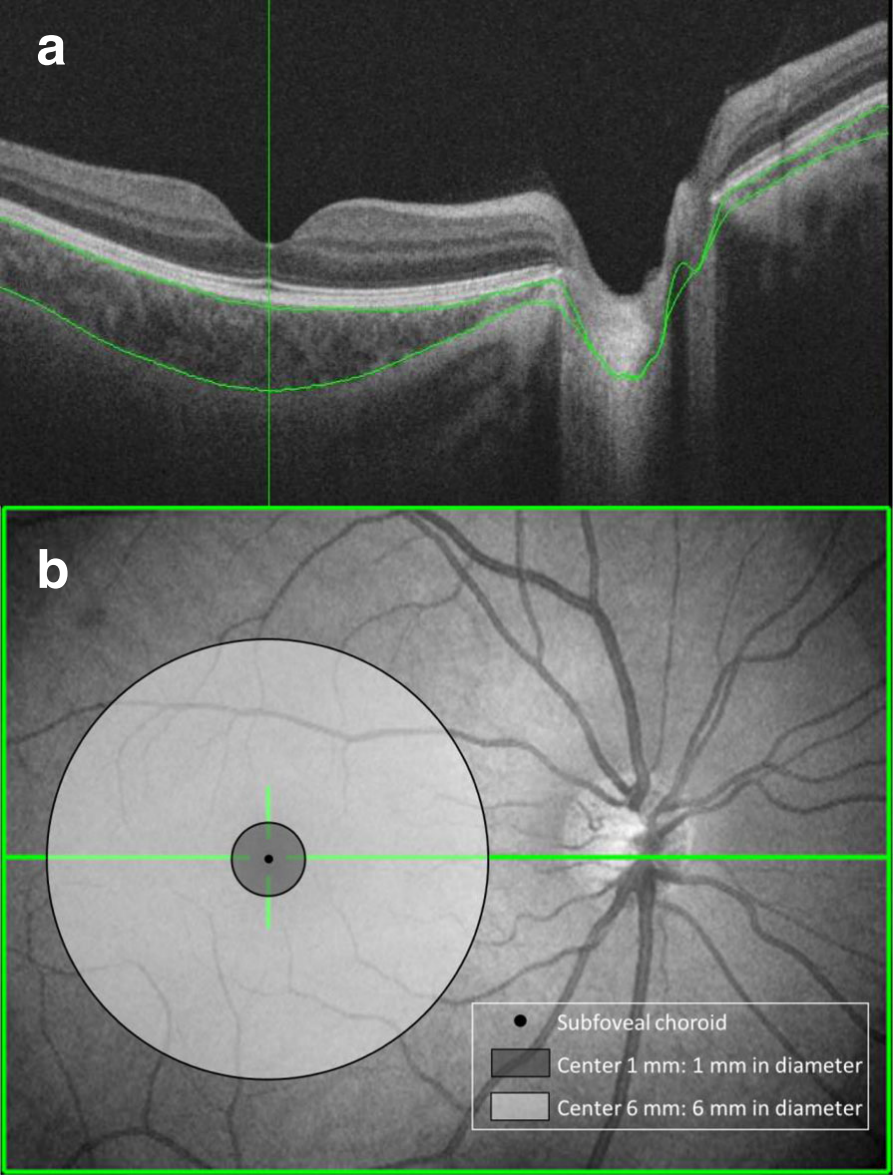
Measurement of choroidal thickness using swept-source optical coherence tomography. **a** The scanning protocol used was the 3D macula with a scan density of 512 × 256 covering a 12 × 9 mm^2^ area. The choroidal thickness was automatically analyzed using built-in tools. **b** The three defined macular regions were: subfoveal choroidal thickness (SFCT), radii of 0.5 mm choroidal thickness (center 1 mm CT), and radii of 3.0 mm choroidal thickness (center 6 mm CT)

The scanning protocol used was the 3D macula with a scan density of 512 × 256 and an area of 12 × 9 mm^2^. After automatically analyzing CT using the built-in tools, the area was divided into three macular regions: subfoveal choroidal thickness (SFCT), radii of 0.5 mm (center 1 mm), and radii of 3.0 mm (center 6 mm) (Fig. 1). Thickness data was corrected for magnification errors through the use of the individual AL, spherical refraction, cylinder refraction, and corneal radius. All SS-OCT examinations were performed by an experienced technician (S.A.) between 9:00 AM and 12:00 PM in order to avoid the inclusion of any diurnal variations in the CT [16].

### Statistical analyses

All statistical analyses were performed using the Bell Curve for Excel version 2.0 software program (Social Survey Research Information Co., Ltd., Tokyo, Japan). Data are presented as the means ± standard deviations. A paired t-test, two sample t-test, and a one-way analysis of covariance (ANCOVA), which was controlled using AL, were used to evaluate the differences between the amblyopic and fellow eyes. CT differences before and after treatment were compared by ANCOVA, which was controlled using AL. Pearson’s correlation coefficient was used to evaluate correlations in logMAR changes versus CT changes after amblyopia treatment. A *p*-value of less than 0.05 was considered to be statistically significant.

## Results

### Demographic data

Initially, 16 Japanese patients with hypermetropic anisometropic amblyopia were enrolled in this study. In three patients the auto-segmentation of the choroid from the sclera was incorrect and SS-OCT image quality was poor, so they were excluded from the study. Therefore, our current study analyzed a total of 13 patients. Table 1 shows the demographic and clinical data of the patients. The mean age of the patients at the enrollment visit was 6.2 ± 2.4 years (range: 4.0 to 11.3 years). The average observation period was 22.2 ± 11.0 months (range: 7 to 36 months). As for the details of the treatment, refractive correction only was performed in four patients, while combined refractive correction and patching was administered in nine patients. The logMAR in amblyopic eyes at the time of enrollment was 0.44 ± 0.27 (range: 1.00 to 0.10), while it was 0.03 ± 0.15 (range: 0.22 to − 0.18) after the treatment. After amblyopia treatment, the logMAR improvement in the amblyopic eyes was 0.41 ± 0.19 (*p* < 0.001). Refraction in the amblyopic eyes was more hyperopic as compared to that for the fellow eyes both before and after the treatment (*p* < 0.001 for both comparisons). The AL in the amblyopic eyes was significantly shorter than that found in the fellow eyes both before and after treatment (*p* < 0.001 for both comparisons). There was a significant extension of the AL in both eyes at the final versus the enrollment visit (amblyopic eyes: 0.33 ± 0.14 mm, *p* < 0.001; fellow eyes: 0.21 ± 0.15 mm, *p* < 0.001).

**Table 1:** Demographic and clinical data of the patients before and after treatment

	Before treatment			After treatment		
	AE (*n* = 13)	FE (*n* = 13)	*p*-value^†^	AE (*n* = 13)	FE (*n* = 13)	*p*-value^†^
Age	6.2 ± 2.4		–	8.1 ± 2.3		–
Sex (Male: Female)	2: 11		–	–		–
Visual acuity (logMAR)	0.44 ± 0.27	− 0.12 ± 0.07	*p* < 0.001*	0.03 ± 0.15	− 0.16 ± 0.05	*p* < 0.001*
Refraction (diopters)	5.58 ± 1.23	2.27 ± 1.43	*p* < 0.001*	5.50 ± 1.39	2.29 ± 1.99	*p* < 0.001*
Axial length (mm)	20.97 ± 0.88	22.11 ± 1.22	*p* < 0.001*	21.31 ± 0.91	22.32 ± 1.25	*p* < 0.001*

*AE*, amblyopic eyes, *FE* fellow eyes

Values are shown as mean ± standard deviation

^†^Two sample t-test; * *p* < 0.01

### CT

Table 2 shows the CT before and after the treatment. SFCT, center 1 mm CT, and center 6 mm CT in the amblyopic eyes were significantly thicker than that in the fellow eyes both before and after the treatment (*p* < 0.05 for all comparisons). There was no significant change in SFCT, center 1 mm CT, and center 6 mm CT seen in either the amblyopic or fellow eye after the treatment.

**Table 2:** CT comparisons before and after treatment in the amblyopic and fellow eyes

	Before treatment		After treatment		*p*-value^‡^			
	AE (*n* = 13)	FE (*n* = 13)	AE (*n* = 13)	FE (*n* = 13)	Before: AE vs. FE	After: AE vs. FE	AE: before vs. after	FE: before vs. after
SFCT (μm)	353.7 ± 86.6	281.1 ± 56.2	336.3 ± 67.9	286.4 ± 57.9	0.013*	0.042*	0.25	0.75
Center 1 mm CT (μm)	352.8 ± 80.1	283.2 ± 54.1	334.6 ± 63.4	285.0 ± 57.3	0.015*	0.043*	0.21	0.84
Center 6 mm CT (μm)	295.1 ± 43.5	259.9 ± 50.7	288.5 ± 36.1	258.8 ± 44.8	0.043*	0.036*	0.84	0.91

*AE* amblyopic eyes, *FE* fellow eyes, *SFCT* subfoveal choroidal thickness, *CT* choroidal thickness

Values are shown as mean ± standard deviation

^‡^ANCOVA using axial length as a covariate; * *p* < 0.05

**Fig. 2:**
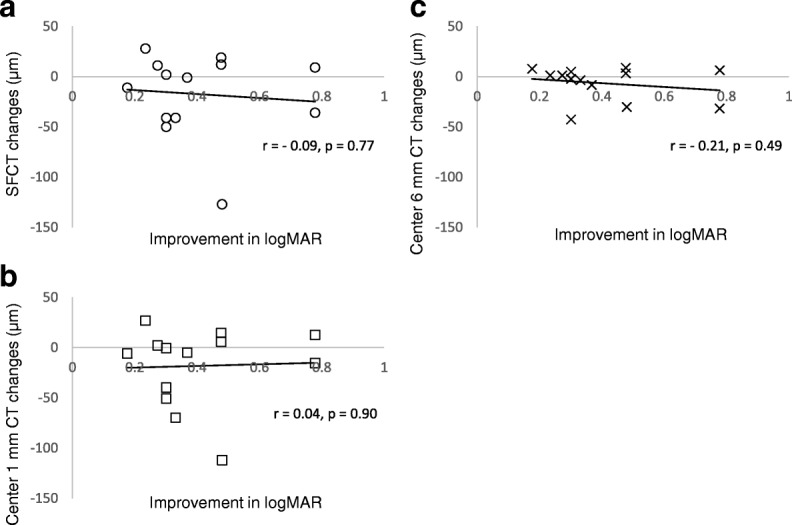
Correlations between logMAR changes versus choroidal thickness changes after treatment in the amblyopic eyes. **a** Scatter plot of improvement in logMAR versus subfoveal choroidal thickness (SFCT) changes. **b** Scatter plot of improvement in logMAR versus center 1 mm choroidal thickness (CT) changes. **c** Scatter plot of improvement in logMAR versus center 6 mm CT changes. The correlations between improvement in logMAR and changes in the SFCT, center 1 mm CT, and center 6 mm CT were not significant

Figure 2 shows that there are not significant correlations between logMAR changes and CT changes in the amblyopic eyes after treatment [SFCT (Fig. 2a): *r* = − 0.09, *p* = 0.77, center 1 mm CT (Fig. 2b): *r* = 0.04, *p* = 0.90, or center 6 mm CT (Fig. 2c): *r* = − 0.21, *p* = 0.49].

## Discussion

Our current study demonstrates that macular CT in the eyes with hypermetropic anisometropic amblyopia, as measured by SS-OCT, was significantly thicker than that in the fellow eyes, similar to that reported by our previous study [10]. In addition, although the treatment significantly improved the VA in the amblyopic eyes, the choroidal thickening of the amblyopic eyes persisted, and there was no significant change found in CT after the amblyopia treatment.

To the best of our knowledge, there have been four previous studies that evaluated CTs before and after amblyopia treatment [11–14]. Öner et al. [11] found no significant differences between the pre- and post-treatment SFCT in the amblyopic and fellow eyes, although they did report that SFCT was larger in the amblyopic versus the fellow eyes before and after treatment. Our results were in agreement with their previous findings. In contrast, Bayhan et al. [12] reported that there was a significant decrease in CT after treatment in amblyopic eyes. In addition, Hashimoto et al. [13] reported that treatment in two anisohypermetropic amblyopia patients resulted in a gradual increase in choroidal blood flow of the macular regions along with an improvement in VA and a decrease in CT. On the other hand, Nishi et al. [14] found that SFCT in eyes with thicker choroid tended to decrease while eyes with a thinner choroid tended to increase in both the amblyopic and fellow eyes after treatment. As the results appear to differ from study to study, there is not yet a consensus regarding CT changes after amblyopia treatment.

There were some differences noted between our current study and the above previous reports. First, the previous studies used a manual analysis to compile CT measurements obtained by SD-OCT [11–14]. However, in our current study, we used SS-OCT, which performs an automatic analysis using a 3D scan. Since our automatic analysis should have evaluated CT more objectively than the manual analysis, differences between the types of analysis used may have led to the disagreement between the results of the previous studies and our current study. Second, the duration of the treatment varied across studies. The studies by Öner et al. and Bayhan et al. evaluated CT at 6 months after starting the treatment [11, 12]. In contrast, we observed the patients after the treatment for a longer term, ranging from 7 to 36 months (mean; 22.2 ± 11.0 months). The difference in the length of the observation period could have had an influence on the degree of the VA improvement. In fact, the improvement of VA in the amblyopic eyes after treatment in our study (logMAR; 0.44 ± 0.27 to 0.03 ± 0.15) was greater than that found by Öner et al. (logMAR; 0.35 ± 0.3 to 0.16 ± 0.2) [11] and Bayhan et al. (Snellen; 0.32 ± 0.2 to 0.74 ± 0.3) [12]. Nevertheless, we did not find any significant change in CT after treatment. Furthermore, our current study also did not find any significant correlation between logMAR changes and CT changes after amblyopia treatment. Nishi et al. [14] showed that the changes in CT were found not only in the amblyopic eye but also in the fellow eye one year after optical correction. Therefore, we believe that the improvement of VA that occurs after the amblyopia treatment does not have a significant effect on CT.

It has been previously reported that an increased macular choroidal thickness correlates with hypermetropia, so in consequence it correlates with a short AL [17, 18]. However, other studies, including our study, show that CT of hypermetropic anisometropic amblyopic eyes is thick, even when the difference in the AL or refractive error between amblyopic and fellow eyes is taken into account in the statistical analysis [10, 12, 19]. Furthermore, other investigations have reported changes in the profile of CT [20], in the choroidal structure [21], and in the choroidal blood flow [13] exist in hypermetropic anisometropic amblyopic eyes. Based on these findings, we assume that there are some structural changes that do occur in the choroid of hypermetropic anisometropic amblyopic eyes. In normal human eyes, it has been reported that SFCT decreases during accommodation [22]. Also, Chakraborty et al. reported that the presence or absence of hyperopic defocus affects the amplitude of the diurnal change in CT [23]. Therefore, these findings suggest that CT might be closely related to the accommodative function and/or hyperopic defocus. On the other hand, it has been reported that amblyopic eyes have accommodative dysfunction compared to fellow eyes for which refractive correction is unnecessary [24]. Therefore, it can be hypothesized that the difference in CT between hypermetropic anisometropic amblyopic eyes and fellow eyes is not entirely due to a short AL, as the accommodative dysfunction and/or hyperopic defocus imposes a secondary effect on the morphology of the choroid in amblyopia. A future study that evaluates the relationship between the accommodative function and CT in amblyopic eyes will need to be undertaken.

The limitation of our present study was the small number of patients examined. A further study that includes a larger number of patients will need to be undertaken in order to definitively confirm the results.

## Conclusion

In conclusion, we found that even though there was a significant improvement of the VA in amblyopic eyes after treatment, the choroidal thickening of amblyopic eyes persisted, and there was no significant correlation between logMAR changes and CT changes after treatment. Therefore, our findings suggest that thickening of CT in amblyopia is not directly related to visual dysfunction.

## Abbreviations

AE: Amblyopic eyes
AL: Axial length
ANCOVA: A one-way analysis of covariance
BCVA: Best-corrected visual acuity
CT: Choroidal thickness
FE: Fellow eyes
logMAR: Logarithm of the minimum angle of resolution
OCT: Optical coherence tomography
SD-OCT: Spectral-domain optical coherence tomography
SFCT: Subfoveal choroidal thickness
SS-OCT: Swept-source optical coherence tomography
VA: Visual acuity
